# High-definition videolaryngoscopy is superior to fiberoptic laryngoscopy: a 111 multi-observer study

**DOI:** 10.1007/s00405-021-06673-0

**Published:** 2021-02-19

**Authors:** Constanze Scholman, Jeroen M. Westra, Manon A. Zwakenberg, Frederik G. Dikkers, Gyorgy B. Halmos, Jan Wedman, Jan E. Wachters, Bernard F. A. M. van der Laan, Boudewijn E. C. Plaat

**Affiliations:** 1 Department of Otorhinolaryngology‐Head and Neck Surgery, University of Groningen, University Medical Center, Groningen Hanzeplein 1, 9700 RB Groningen, The Netherlands; 2 Department of Otorhinolaryngology, Amsterdam UMC, University of Amsterdam, Amsterdam, The Netherlands; 3 Department of Otorhinolaryngology‐Head and Neck Surgery, Haaglanden Medical Center, The Hague, The Netherlands

**Keywords:** Videolaryngoscopy, Laryngeal diseases, Squamous cell carcinoma, Sensitivity, Fiberoptic

## Abstract

**Purpose:**

This study aims to analyse differences in fiberoptic laryngoscopy (FOL) versus high definition laryngoscopy (HDL) by examining videolaryngoscopy images by a large group of observers with different levels of clinical expertise in ear, nose and throat (ENT) medicine.

**Methods:**

This study is a 111 observer paired analysis of laryngoscopy videos during an interactive presentation. During a National Meeting of the Dutch Society of ENT/Head and Neck Surgery, observers assessed both FOL and HDL videos of nine cases with additional clinical information. Observers included 41 ENT consultants (36.9%), 34 ENT residents (30.6%), 22 researchers with Head and Neck interest (19.8%) and 14 with unspecified clinical expertise (12.6%). For both laryngoscopic techniques, sensitivity, specificity, positive and negative predictive value and diagnostic accuracy were determined for identifying a normal glottis, hyperkeratosis, radiotherapy adverse effects and squamous cell carcinoma. The sensitivities for FOL and HDL were analysed with regard to the different levels of clinical expertise.

**Results:**

The overall sensitivity for correctly identifying the specific histological entity was higher in HDL (FOL 61% vs HDL 66.3%, *p* < 0.05). HDL was superior to FOL in identifying a normal glottis (FOL 68.1% vs HDL 91.6%, *p* < 0.01) and squamous cell carcinoma (FOL 70.86% vs HDL 79.41%, *p* = 0.02). Residents and researchers with Head and Neck interest diagnosed laryngeal lesions more correctly with HDL (*p* < 0.05).

**Conclusions:**

In a large population of observers with different levels of clinical expertise, HDL is superior to FOL in identifying laryngeal lesions.

## Introduction

Laryngeal visualisation is the most important diagnostic tool for lesions of the larynx [1]. For timely detection and improved survival, it is essential to differentiate between a benign and a malignant lesion in an early stage [2]. Nowadays, various techniques to visualize the larynx are used in daily clinical practice including fiberoptic laryngoscopy (FOL) and high-definition videolaryngoscopy (HDL). Since the introduction of FOL in 1954 as an alternative to a mirror examination, FOL is used on a worldwide scale [3, 4]. However, the quality of the images obtained with FOL might make it hard to distinguish a malignant from a benign lesion and minor epithelial changes could be overlooked [5]. HDL, which was developed after the introduction of digital chip-on tip endoscopy in 1983, shows a superior image quality and has the potential to improve diagnostics of laryngeal lesions [6, 7]. In a previous study, we found that HDL is superior to FOL in detecting mucosal abnormalities and enhanced the level of diagnostic accuracy [7]. In that study, observers with at least 5 years of clinical experience in the field of laryngology and/or Head and Neck oncology examined pharyngeal and laryngeal videos recorded by both FOL and HDL. We could not contribute the improvement in detection completely to HDL, because clinical experience and a developed reliable sense of intuition improve diagnostic decisions as well [8]. Based on the dual-process theory, which distinguishes between analytic and non-analytic knowledge, experts have gained analytic knowledge which acquires critical thinking and hypothetical and counterfactual reasoning [8]. Non-analytic knowledge depends on intuitiveness, unconsciousness and an automatic pattern recognition [8]. Beginners particularly use the non-analytic knowledge for the diagnostic process [9]. To confirm earlier conclusions regarding the superiority of HDL compared to FOL from a group of well-selected highly experienced observers to a more diverse population of observers, laryngeal videos were examined by 111 observers with a wide range of clinical expertise on a National Meeting of the Dutch Society of Otorhinolaryngology/Head and Neck Surgery.

## Materials and methods

### Ethical considerations

The institutional Review Board of the University Medical Center Groningen concluded that this retrospective study with anonymised videolaryngoscopies did not fall under the scope of the Dutch Medical Research Involving Human Subjects Act (WMO).

### Patients and procedure

**Fig. 1:**
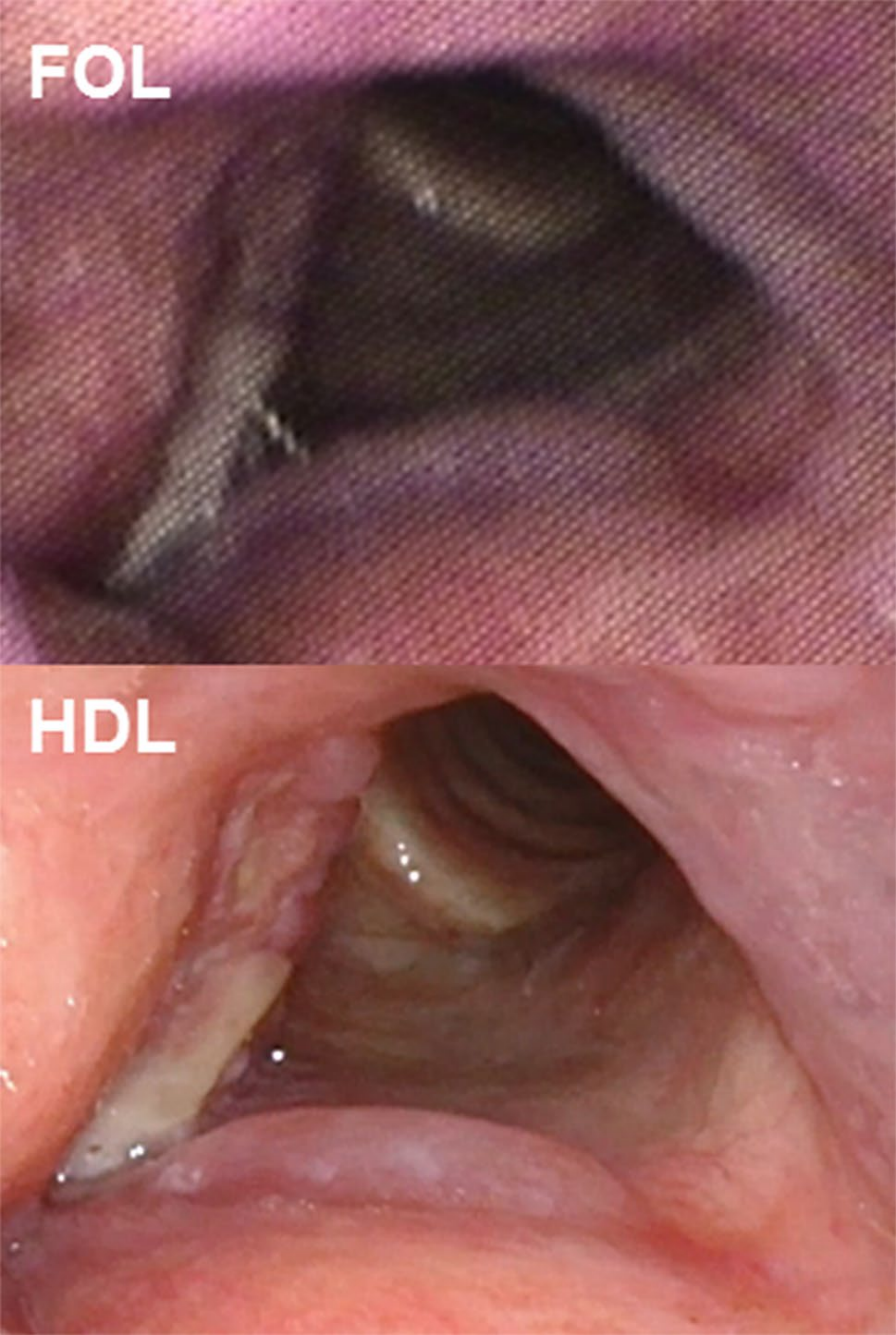
Representative picture of squamous cell carcinoma of anterior part of right vocal fold, as captured with FOL and HDL. FOL, fiberoptic laryngoscopy; HDL, high-definition laryngoscopy

In total, nine laryngeal lesions were included from our database of pharyngeal and laryngeal endoscopic recordings of 51 patients. The database was composed during routine diagnostic procedures between June 2014 and October 2017, as reported previously [6]. The FOL and HDL video had to be recorded without treatment between each recording. The median time frame between the FOL and HDL video recording was 3 days with a standard deviation of 97 days. Examples applied in our study group are shown in Fig. 1. For the recordings, we used a flexible fiberoptic laryngoscope ENF-GP (Olympus Medical Systems, Tokyo, Japan) linked to a Matrix E camera processer (Xion Gmbh, Berlin, Germany) for FOL and a flexible video rhinolaryngoscope ENF VH (Olympus Medical Systems, Tokyo, Japan) attached to a HD monitor for HDL. After the patients’ informed consent, videos, patient data and histopathological results from the electronic patient records were collected. Only glottic lesions were included when there was both one FOL and one HDL video available from the same lesion without treatment between the recordings. We had to limit the number of diagnoses to four categories to ensure the possibility of presenting them to the observers with the survey tool “Kahoot!”, which allows a maximum amount of four answer options [10]. The following four entities were included: normal, hyperkeratosis, radiotherapy adverse effects and squamous cell carcinoma. Hyperkeratosis and squamous cell carcinoma were confirmed after histological examination. Normal glottis and radiotherapy adverse effects was a clinical diagnosis without histology. We included at least videos of two patient’s lesions per diagnosis to receive a more reliable result.

All videos were arranged into an interactive presentation with a time limit of 15 min in total. The introduction and instruction of the audience were calculated with a time span of 5 min, and each video was shown within 30 s. In this way, there were 10 min available for presenting the videos. In total, nine cases were included resulting in 18 videos by showing one FOL and one HDL video in a random order. In 11 of the 18 videos, the vocal folds were shown as well in open position for breathing as in closed position during voicing. In seven of the 18 videos, the vocal folds were shown in an open position for breathing only but showed similar small movements during breathing.

Using Windows Movie Maker 2012 (Microsoft Corp, Redmond, WA, USA), videos were edited into fragments of 10 s. Narrow-band imaging recordings were excluded from the HDL videos. Moreover, both videos from the same lesions showed the lesion soundless from a similar distance. We have built a PowerPoint 2010 (Microsoft Corp, Redmond, WA, USA) presentation in Dutch including each video with additional patient information (gender, age, intoxications, medical history) in a randomised order. The additional patient information included gender, age, intoxications like smoking in pack-years and alcohol use per week. Also, medical history of ENT diseases was given. For the diagnosis radiotherapy adverse effects information was presented about the time span between the last given radiotherapy dose and the recorded video and the tumor stage of the previous tumor. During a biannual national meeting of the Dutch Society of ENT/Head and Neck Surgery (Nieuwegein, Netherlands, 22nd Nov 2018), the videos were presented interactively to a Dutch audience. The presentation was presented in a parallel session dedicated to Head and Neck oncology. After a 5 min introduction containing explanations about the survey tool, participants logged in with their smartphones on the website “https://kahoot.it/” with a presented password and an anonymous user name [10]. The first question was focusing on the different level of clinical expertise. Observers had to indicate which category suited best: ENT surgeon, ENT resident, researcher or other. Missing information was counted into the group “other”. Thereafter, every question started with an introduction of the relevant medical history followed by showing the corresponding video. For each video observers had to select the most likely histological entity.

### Statistical analysis

SPSS version 22.0 (IBM Corp., Armonk, NY, USA) was used for calculating sensitivity, specificity, positive predictive value (PPV) and negative predictive value (NPV) and diagnostic accuracy for each diagnosis for FOL and HDL separately. For statistical analysis, the answers which were left blank were excluded. The chi-square test was used to analyse differences between both laryngoscopes. A *p* value < 0.05 was considered statistically significant.

## Results

### Patients

In this study, laryngeal lesions of eight men and one woman were included (average age 71 years, 9.2 SD, range: 60–84 years). Almost half of the patients had no previous ENT medical history (44.4%). One third had received radiotherapy due to previous laryngeal malignancy (33.3%).

### Observers

Altogether, 111 observers participated in the interactive presentation which resulted in 1787 observations. In total, 41 ENT consultants (36.9%), 34 ENT residents (30.6%) and 22 researchers with Head and Neck interest (19.8%) participated. In 14 observers, the level of clinical expertise was unspecified (12.6%).

The overall sensitivity for correctly identifying the specific histological entity was higher in HDL (FOL 61% vs HDL 66.3%, *p* < 0.05).

### Identifying a normal glottis and diagnosing benign lesions

As demonstrated in Table 1, a normal glottis was correctly identified in 68.1% (FOL) and 91.6% (HDL) (*p* < 0.01). HDL’s specificity (92.7%) and PPV (74.5%) were higher than FOL’s (89.8, 65.9%, respectively), however, not significantly different. The NPV and accuracy were higher during HDL (FOL 90.7 and 85% vs HDL 97.9 and 92.5%, respectively, *p* < 0.01).

**Table 1:** Sensitivity, specificity, PPV, NPV and accuracy of stating the entities normal glottis and squamous cell carcinoma

	Entity: normal glottis			Entity: squamous cell carcinoma		
Videos	FOL (%) (n)	HDL (%) (n)	*χ*^2^ (*p* value)*	FOL (%) (n)	HDL (%) (n)	*χ*^2^ (*p* value)*
Sensitivity	68.14 (139 of 204)	91.57 (152 of 166)	29.92 (< **0.01**)	70.86 (214 of 302)	79.41 (243 of 306)	5.95 (**0.02**)
Specificity	89.80 (643 of 699)	92.69 (659 of 711)	3.69 (0.06)	86.51 (526 of 608)	85.64 (489 of 571)	0.19 (0.66)
PPV	65.88 (139 of 211)	74.51 (152 of 204)	3.69 (0.55)	72.30 (214 of 296)	74.77 (243 of 326)	0.49 (0.49)
NPV	90.70 (634 of 708)	97.92 (659 of 673)	32.25 (< **0.01**)	85.67 (526 of 614)	88.59 (489 of 552)	2.20 (1.39)
Accuracy	84.95 (782 of 910)	92.47 (811 of 877)	19.74 (< **0.01**)	81.32 (740 of 910)	83.47 (732 of 877)	1.42 (0.23)

Bold values are indicate *p* < 0.05

*PPV* positive predictive value, *NPV* negative predictive value, *FOL* fiberoptic laryngoscopy, *HDL* high-definition laryngoscopy, *Chi-square test

For the detection of hyperkeratosis and radiotherapy adverse effects, there was no difference between FOL and HDL (*p* > 0.05, Table 2). HDL showed a higher specificity compared to FOL in both hyperkeratosis and radiotherapy adverse effects (FOL 86.9 and 92.4% vs HDL 90.4 and 97.6%, *p* < 0.05). The accuracy of detecting hyperkeratosis did not differ between both laryngoscopes (FOL 82.4% vs HDL 84.6, NS) whereas it varied in stating radiotherapy adverse effects (FOL 85.2% and HDL 90.4, *p* < 0.01).

**Table 2:** Sensitivity, specificity, PPV, NPV and accuracy of stating the entities hyperkeratosis and radiotherapy adverse effects

	Entity: hyperkeratosis			Entity: radiotherapy adverse effects		
Videos	FOL (%) (n)	HDL (%) (n)	*χ*^2^ (*p* value)*	FOL (%) (n)	HDL (%) (n)	*χ*^2^ (*p* value)*
Sensitivity	66.50 (133 of 200)	65.52 (133 of 203)	0.04 (0.84)	60.29 (123 of 204)	66.34 (134 of 202)	1.60 (0.21)
Specificity	86.90 (617 of 710)	90.36 (609 of 674)	4.08 (**0.04**)	92.35 (652 of 706)	97.63 (659 of 675)	19.98 (< **0.01**)
PPV	58.85 (133 of 226)	67.17 (133 of 198)	3.13 (0.08)	69.49 (123 of 168)	89.33 (134 of 150)	19.00 (< **0.01**)
NPV	90.2 (617 of 684)	89.69 (609 of 679)	0.10 (0.75)	88.95 (652 of 733)	90.65 (659 of 727)	1.15 (0.28)
Accuracy	82.42 (750 of 910)	84.61 (742 of 877)	1.55 (0.21)	85.16 (775 of 910)	90.42 (793 of 877)	19.76 (< **0.01**)

Bold values are indicate *p* < 0.05

*PPV* positive predictive value, *NPV* negative predictive value, *FOL* fiberoptic laryngoscopy, *HDL* high-definition laryngoscopy, *Chi-square test

### Detecting squamous cell carcinoma

**Fig. 2:**
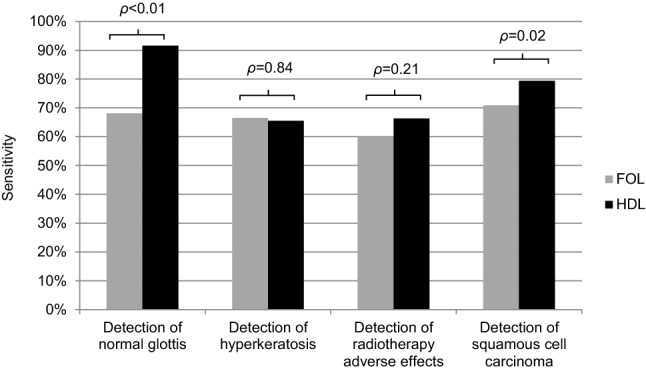
Overview of sensitivities for stating the histological entities normal glottis, hyperkeratosis, radiotherapy adverse effects and squamous cell carcinoma (all 111 observers). Bars: grey: fiberoptic laryngoscopy; black: high-definition laryngoscopy

As shown in Table 1, the sensitivity of detecting a laryngeal malignancy using HDL was 8.6% higher compared to FOL (FOL 70.9% vs HDL 79.4%, *p* = 0.02). There were no differences in specificity, PPV, NPV and accuracy (*p* > 0.05). An overview of the sensitivities for every diagnosis is provided in Fig. 2.

### Sensitivity for FOL and HDL by the level of clinical expertise

Table 3 demonstrates the sensitivity for FOL and HDL separately for each level of clinical expertise. The ENT consultants and observers with an unspecified clinical expertise showed no significant difference in sensitivity of FOL compared to HDL. The ENT residents and researchers detected the specific histological entity more accurately using HDL (*p* < 0.05).

**Table 3:** Sensitivity for diagnosing a normal glottis, hyperkeratosis, radiotherapy adverse effects and squamous cell carcinoma for each level of clinical expertise

	Sensitivity		
Level of clinical expertise	FOL (%) (n)	HDL (%) (n)	*χ*^2^ (*p* value)*
ENT surgeon (*n* = 41)	72.9 (237 of 325)	73.6 (231 of 314)	0.03 (0.85)
ENT resident (*n* = 34)	64.8 (188 of 290)	80.7 (222 of 275)	17.92 (< **0.01**)
Researcher (*n* = 22)	61.6 (114 of 185)	71.8 (130 of 181)	4.29 (**0.04**)
Other (*n* = 14)	63.64 (70 of 110)	73.83 (79 of 107)	2.62 (0.11)

Bold values are indicate *p* < 0.05

*FOL* fiberoptic laryngoscopy, *HDL* high-definition laryngoscopy, *Chi-square test

## Discussion

### Key findings

To the best of our knowledge, this is the first study in which FOL is compared to HDL in a large group of observers with a wide range of clinical expertise. This study showed that the overall sensitivity for correctly identifying the specific histological entity is higher in HDL (FOL 61% vs HDL 66.3%, *p* < 0.05). HDL is superior to FOL in identifying a normal glottis (FOL 68.1% vs HDL 91.6%, *p* < 0.01) and squamous cell carcinoma (FOL 70.86% vs HDL 79.41%, *p* = 0.02). Furthermore, this study demonstrated that ENT residents and researchers with Head and Neck interest identify laryngeal lesions more accurately using HDL compared to FOL.

### Identifying a specific histological entity

Our results strengthen the reliability that diagnostic accuracy for HDL is higher than sensitivity obtained with FOL. HDL is superior in identifying a normal glottis. In FOL, 25% of the observers judged the normal glottis inaccurately as hyperkeratosis and in HDL this was 6.6%. The superiority of HDL to detect a normal glottis is an improvement since it was not shown in our previous study [7]. An explanation could be the large number of observations. For evaluation of a normal glottis with both endoscopes, we reached an amount of more than 200 observations versus 18 observations in the previous study [7]. In this way, we were able to manifest slight differences into significant ones.

The higher detection rate with HDL of squamous cell carcinoma is in line with our previous study and is of great importance for a patient presenting with a malignant lesion of the glottis [7, 10]. Early detection of malignancy has an essential impact on the prognosis of the patient [2]. A reason for the superior sensitivity of HDL might be the improved image quality of HDL [7, 11, 12].

### The level of clinical expertise

The diagnostic values of HDL showed superiority not only in highly experienced observers in our previous study but also in observers with a wide range of clinical expertise [7]. The sensitivity increases in 15% of the cases when ENT residents, who are by definition less experienced than ENT consultants, are using HDL instead of FOL [13]. This can influence decision-making in daily patient care. Furthermore, researchers with Head and Neck interest showed a higher sensitivity using HDL. It is difficult to define the stage of clinical expertise of the researchers because we do not know their specific research topic, although due to the meeting’s nature presumably all of them were performing Head and Neck-related research. However, the improved sensitivity might be explained by the improved image quality of HDL for both ENT-residents and researchers. In literature, HDL showed a high image resolution and is able to clearly show small lesions on the mucosa [11, 12]. This could support pattern recognition which is particularly used in the non-analytical knowledge in beginners, i.e. the ENT-residents [8, 9]. Consequently, a more accurate identification of the laryngeal lesion is achievable. Surprisingly, the sensitivity of ENT-residents was higher compared to the sensitivity of the ENT-consultants. ENT-consultants might be specialized in rhinology or otology which results in less clinical experience in laryngoscopy. Furthermore, ENT-residents might be more used to HDL than ENT-consultants due to HDL experience in their clinical education. Interestingly, the sensitivity of ENT consultants did not differ between FOL and HDL. This might be explained by the fact that these observers are highly experienced in recognizing laryngeal lesions. In experts, the diagnostic accuracy is less influenced by the image quality of the diagnostic tool but also by the analytical knowledge [8].

### Strengths of the study

For the first time, we used an interactive presentation to reach a large group of 111 observers with different levels of clinical expertise. The methodology of a paired analysis (i.e., FOL and HDL of the same glottis) in a randomised order with 111 observers provided us with a very large amount of data (1787 observations) allowing solid statistical analysis. Furthermore, we were able to compare the results with our previous study result due to comparable variables and study design [7]. The added clinical information and using videos instead of photos allow us to draw conclusion which is useful in daily clinical practice.

### Limitations of the study

All observers had to perform the survey tool during an interactive presentation in one congress hall at the same time. First, we cannot exclude that some observers were discussing the histological entity during the 30 s. Second, the percentage of dedicated survey participants compared to the total audience is unknown. Hence, a voluntary response bias might have influenced the survey results. Also, the survey tool offered a maximum number of four answer options resulting in the inclusion of four different histological entities. Consequently, we are not able to apply our results to a wider range of diagnoses like metaplasia, cysts, inflammation, mild or severe dysplasia. Still, for assessing videos of laryngoscopy and inter-observer variability, an interactive software, such as the survey tool “Kahoot!”, offers great possibilities to receive a large amount of data within a short period of time. Furthermore, we did not include Narrow Band Imaging or Stroboscopic recordings because we wanted to focus on the comparison of FOL with HDL. Highlighting the focus between the two variables aimed to attain a higher audience attention. Lastly, showing soundless videos of laryngeal lesions are controversial because the clinical diagnosis can be supported by the examination of voice performance. Nevertheless, visual examination of the glottis is of great importance for the diagnosis [14, 15].

### Clinical applicability of the study

This study shows that HDL is not only a tool for highly experienced experts but also for colleagues with a wide range of clinical expertise who have to assess laryngeal lesions. HDL improves diagnostic accuracy in the daily clinical setting.

## Conclusion

Using a large set of data, gathered in an interactive presentation, we were able to demonstrate an advantage of HDL to FOL to identify laryngeal lesions with videolaryngoscopy by observers with a wide range of clinical expertise. This study confirms earlier conclusions regarding the superiority of HDL compared to FOL from a group of well-selected highly experienced observers to a more general population working in the field of ENT.

## Data Availability

The datasets generated during the current study are available from the corresponding author on reasonable request.
